# Maximal sustainable energy intake during transatlantic ocean rowing is insufficient for total energy expenditure and skeletal muscle mass maintenance

**DOI:** 10.1113/EP091319

**Published:** 2023-11-15

**Authors:** Rosalind K. Holsgrove‐West, Raquel Revuelta Iniesta, Doaa R. Abdelrahman, Andrew J. Murton, Benjamin T. Wall, Francis B. Stephens

**Affiliations:** ^1^ Public Health and Sport Sciences, Medical School University of Exeter Exeter UK; ^2^ Department of Surgery University of Texas Medical Branch Galveston Texas USA

**Keywords:** atrophy, doubly labelled water, energy expenditure endurance, energy intake, muscle maintenance, ocean rowing

## Abstract

Studies of extreme endurance have suggested that there is an alimentary limit to energy intake (EI) of ∼2.5 × resting metabolic rate (RMR). To gain further insight, this study aimed to simultaneously measure EI, total energy expenditure (TEE) body mass and muscle mass in a large cohort of males and females of varying ages during a transatlantic rowing race. Forty‐nine competitors (m = 32, f = 17; age 24–67 years; time at sea 46 ± 7 days) in the 2020 and 2021 Talisker Whisky Atlantic Challenge rowed 12–18 hday^−1^ for ∼3000 miles. TEE was assessed in the final week of the row using ^2^H_2_^18^O doubly labelled water, and EI was analysed from daily ration packs over this period. Thickness of relatively active (vastus lateralis, intermedius, biceps brachaii and rectus abdominus) and inactive (gastrocnemius, soleus and triceps) muscles was measured pre (<7 days) and post (<24 h) row using ultrasound. Body mass was measured and used to calculate RMR from standard equations. There were no sex differences in males and females in EI (2.5 ± 0.5 and 2.3 ± 0.4 × RMR, respectively, *P* = 0.3050), TEE (2.5 ± 1.0 and 2.3 ± 0.4 × RMR, respectively, *P* = 0.5170), or body mass loss (10.2 ± 3.1% and 10.0 ± 3.0%, respectively, *P* = 0.8520), and no effect of age on EI (*P* = 0.5450) or TEE (*P* = 0.9344). Muscle loss occurred exclusively in the calf (15.7% ± 11.4% *P* < 0.0001), whilst other muscles remained unchanged. After 46 days of prolonged ultra‐endurance ocean rowing incurring 10% body mass loss, maximal sustainable EI of ∼2.5 × RMR was unable to meet total TEE suggesting that there is indeed a physiological capacity to EI.

## Abstract (highlights)

- **What is the central question of this study?**
  During a prolonged endurance transatlantic ocean rowing race over ∼46 days, what changes occur in energy intake (EI), total energy expenditure (TEE) and body mass and are there differences between males and females or an effect of age?
- **What is the main finding and its importance?**
  During a ∼46 days, there appeared to be a maximal sustainable EI of ∼2.5 × resting metabolic rate (RMR), matching TEE of 2.5 × RMR, incurring a 10% body mass loss There were no sex differences, and minimal influence of age.

## 1 INTRODUCTION

Prolonged endurance events with high daily total energy expenditures (TEE) provide a unique opportunity to study the physiological limits to human performance. When nutritional energy availability is adequate, body mass maintenance during prolonged endurance events is directly related to the physical activity level (Cooper et al., 2011). Physical activity level is a ratio of TEE to basal metabolic rate (BMR) and its range is referred to as metabolic scope (MS). There is a general consensus in the literature that the ceiling for sustainable metabolic scope (SMS) in humans, that is, where no body mass loss occurs over a prolonged period, is around 2.5 × BMR for the general population (Cooper et al., 2011; Westerterp, 2010). Increased energy demand through years of consistent high‐level training can increase this value up to 5 × BMR in athletes, but only in endurance events lasting up to a few weeks (Cooper et al., 2011; Westerterp et al., 1986). When the duration of the endurance activity is extended beyond a few weeks, it appears that SMS decreases curvilinearly eventually plateauing at around 2.5 × BMR (Thurber et al., 2019). Indeed, three runners participating in a 20‐week transcontinental race across America averaged 3.1 × BMR but lost 3.7 kg (5%) in body mass (Thurber et al., 2019), presumably from fat and inactive muscle tissue.

Interestingly, it has been estimated from doubly labelled water measurements of TEE during extreme endurance events and overfeeding studies that a value of around 2.5 × BMR also appears to be the alimentary limit (i.e., food ingestion and/or caloric absorption) to energy intake (EI) (Thurber et al., 2019). Elite endurance athletes have shown they can overcome this ceiling by training their gastrointestinal system and by frequently ingesting high‐carbohydrate drinks and snacks to achieve up to 5 × BMR (Brouns et al., 1989; Westerterp et al., 1986) but this is rarely consistent beyond a few days. During the three weeks of the Tour de France, cyclists favour a cyclical pattern, increasing and decreasing EI depending on stages, and over 3 weeks are able to match EI with TEE (Saris et al., 1989). To our knowledge, this EI limit to SMS in humans has never been tested experimentally in prolonged (>3 weeks) endurance events or in recreational athletes.

Previous endurance studies have suggested that there may be an impairment in EI in older cyclists (Rosenkilde et al., 2015), but they were not compared to younger controls and were only tested over 2 weeks. Similarly, it was suggested that younger males had a higher relative EI than females over an Antarctic crossing, but they were not tested on the same expedition or under the same conditions (Hattersley et al., 2020). Thus, it is currently not known whether EI or SMS differs with age or biological sex. Moreover, of the studies where SMS has exceeded EI, composition of body mass loss is rarely reported and it is unclear whether there is a preferential preservation of physically active muscle mass, as previously observed in events lasting up to a few weeks (Rosenkilde et al., 2015).

The Talisker Whisky Atlantic Challenge is an annual 3000‐mile rowing race across the Atlantic Ocean from La Gomera, Canary Islands to Antigua. Male and female recreational athletes of varying ages who have trained for at least a year compete either solo or as teams, rowing for between 8 and 18 h per day in a continuous 2–3 h on/off pattern for between 30 and 70 days. There is an adequate supply of food to consume as rowers must take a minimum of 60 kcal kg body mass^−1^ day^−1^ (i.e., at least 3 × BMR per person) as a requirement of the race. The emergence and accessibility of such an event provides a unique opportunity to provide further insight as to whether an EI limit to SMS exists.

Therefore, the aim of this study was, for the first time, to simultaneously measure TEE, EI and body mass in a large cohort of males and females of varying ages, during a prolonged endurance ocean rowing race. Assuming an EI limit of 2.5 × BMR, and observations of around 10% body mass loss in previous transatlantic races, it was hypothesised that TEE is likely to be high and ∼5000–7000 kcal day^−1^ (i.e., approximately 3–3.5 × BMR per person). Secondary aims were to investigate whether any sex and age differences exist in the physiological response to prolonged endurance and to characterise changes in any body mass loss by measuring body composition and thickness of various muscles.

## 2 METHODS

### 2.1 Ethical approval

This study was approved by the University of Exeter, College of Life and Environment Sciences, Sport and Health Science Research Ethics Committee (Reference numbers: 201021‐A‐08 and 21‐10‐20‐A‐05) and was conducted in accordance with the *Declaration of Helsinki* (last modified in 2013) and registered at clinicaltrials.gov (NCT05729841). Written informed consent from all volunteers was obtained prior to participation in the study.

### 2.2 Participants

Fifty‐six healthy rowers were recruited from the 2020 and 2021 Talisker Whisky Atlantic Challenge races. All participants had undergone individual training programmes consisting of weekly rowing, mobility, strength and conditioning sessions, exercising for 4–6 days per week in the year preceding the race. Each participant had undergone a medical assessment according to race entry rules and was deemed fit to race. Exclusion criteria were any form of neurological, metabolic, gastrointestinal or musculoskeletal disease, and any below‐knee amputees.

### 2.3 General protocol

The pre‐post experimental protocol was conducted on a one‐off visit at two locations (start and finish). With permission from the race organiser, participants were given the opportunity to take part in the experiment at race registration at the pre‐race village in San Sebastien, La Gomera, Spain and reported to the race tent within 7 days of the race start for a series of measurements. Post‐race measurements were conducted in an identical manner, within 24 h of making landfall, in the post‐race village in English Harbour, Antigua.

In 2020, 27 participants underwent measurements of TEE using the doubly labelled water method, EI analysis, body mass, and ultrasound muscle thickness (*T*_m_) of the gastrocnemius medialis (GM) and soleus (SOL) and blood sampling. One participant was excluded for admitting that the diet in the final week was not representative of the rest of the row. The TEE urine samples for a second participant were believed to be contaminated on arrival, with Eppendorf tubes having clicked open. These samples were discarded and the participant was excluded from analyses. In 2021, 30 participants underwent body mass and ultrasound measurements of the GM, SOL, vastus lateralis (VL), vastus intermedius (VI), rectus abdominis (RA), biceps brachii (BB) and triceps brachii (TB). Five participants were lost to follow‐up due to logistical reasons and one participant did not complete the race. GM was further analysed for muscle architecture by measuring pennation angle (PA) and fascicle length (*L*_f_). The two years of GM data were compared and pooled for analysis.

### 2.4 Energy intake

Energy intake was calculated in two ways. Firstly, it was measured from ration packs. Rowers were asked to maintain a similar daily eating pattern throughout the row, eat as much as possible from their ration pack and to keep all uneaten food and packaging for the final five days in the same daily pack. Daily ration packs for these final five days were analysed; remaining food was weighed and total EI and macronutrient content calculated (Nutritics, 2022). A race food plan was provided by the rowers, pre‐race, outlining all the meals, brands and snacks, fulfilling the minimum race requirement of 60 kcal kg^−1^ day^−1^. A post‐row questionnaire was used to identify any systematic daily omissions or unusual habits whilst at sea and use of supplements and medications. Secondly, EI was calculated as TEE minus energy equivalence of body composition change (body mass loss) using the chemical energy equivalence of fat mass (39.5 MJ kg^−1^) and fat free mass (7.6 MJ kg^−1^) (Hall, 2008), assuming changes in energy stores reflect energy balance during this period (Rosenkilde et al., 2015). As body mass changes were reflective of the entire row, whilst TEE (from doubly labelled water) and EI (ration pack) were from the final week, comparison of both methods enabled a more accurate representation of EI.

### 2.5 Energy expenditure: doubly labelled water

TEE was measured using the doubly labelled water (DLW) two‐point method in the final week of the row. According to the ‘Maastricht protocol’ with calculations presented elsewhere (Speakman et al., 2021; Westerterp et al., 1995), a dose of 0.09 g kg^−1^ of 10% ^18^O and 0.18 g kg^−1^ of 70% ^2^H_2_ (CK Isotopes Ltd, Newtown Unthank, UK) based on pre‐row BM was provided, along with three urine sampling containers. When participants were thought to be approximately 1 week from making landfall, it was communicated via satellite phone call to take a baseline (*T*_0_) urine sample (to establish background isotope enrichment) followed by labelled water consumption. After labelled water consumption, rowers were instructed to rinse the container and consume the remaining water to ensure total dose ingestion. The following morning participants were reminded to collect a second urine sample (*T*_1_) and then reminded again on the day of arrival (*T*_2_) prior to stepping on land. Exact time and date of samples was recorded. *T*_1_ and *T*_2_ were used to measure the isotope elimination rates. The urine samples were collected upon arrival, filtered, centrifuged (10 min, 1,538 *g*; Hettich EBA 200, Hettich GmbH % Co, KG, Tuttlingen, Germany), and immediately frozen at −20°C in Antigua. Once the final participant had completed the race samples were transferred and stored at −80°C at the American University of Antigua until shipment, via the UK, to the University of Texas Medical Branch for isotope enrichment analysis. Analysis was performed in duplicate, using gas chromatography isotope ratio mass spectrometry (Thermo Fisher Scientific Delta V Advantage, Bremen, Germany) equipped with a Finnigan GasBench II (Thermo Fisher Scientific, Waltham, MA, USA). Standard calibration curves were prepared using 99.9% deuterium‐enriched water (Sigma‐Aldrich, St Louis, MO, USA) and 10% ^18^O‐enriched water for measuring enrichment of ^2^H_2_ and ^18^O, respectively.

Background enrichments were subtracted from *T*_1_ and *T*_2_. Dilution spaces (*N*_D_ and *N*_O_) from the least squares regression of ln *T*_1_ and ln *T*_2_ were calculated via the two‐point slope method and extrapolated to time zero. Elimination rates *k*_D_ and *k*_O_ were measured from the same regression. It is assumed that dilution spaces did not appreciably change during the last week, which is generally considered consistent within 10 days (Wolfe & Chinkes, 2004).

**FIGURE 1:**
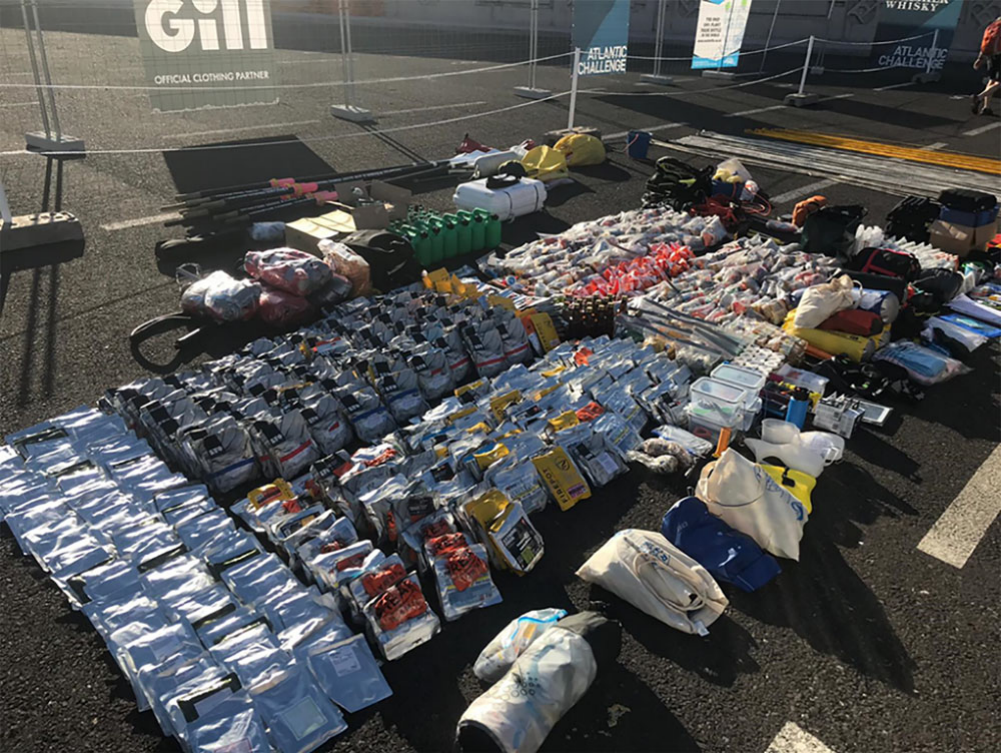
Daily ration and snack packs for a typical crew of four people. Individualised ration packs in the foreground are taped into daily units. Crews of four are required to take a minimum of 55 days of rations, solos are required to take a minimum of 85 days. Each rowers is required to take a minimum of 60 kcal kg^−1^ day^−1^.

Rate of CO_2_ production was measured using the following equation (Speakman et al., 2021):

(1) $$rCO_{2}\left(litresday^{-1}\right)=0.4554N\left[\left(1.007k_{O}\right)-\left(1.043k_{D}\right)\right]22.26$$

where *k*_D_ and *k*_O_ are in units of day^−1^, *N* is in moles, and *r*CO_2_ is in litres day^−1^. TEE was estimated using the following equation (Weir, 1949):

(2) $$TEE\left(MJday^{-1}\right)=\left[rCO_{2}\left(1.106+\left(3.94/FQ\right)\right)\times \left(4.184/10^{3}\right)\right]$$

where FQ is the food quotient calculated from daily energy intake (Figure 1). Given the pre‐packed ration packs on board the boat and no access to other foods, it is assumed that ration pack content did not change considerably whilst as sea. Energy content of four separate ration packs was verified using bomb calorimetry (1341 Plain Jacket, Parr Instrument Company, Moline, IL, USA) where each meal was rehydrated, homogenized and samples measured in duplicate, averaged and found to be consistent with food labelling. Student's *t*‐test showed no difference between food label and bomb calorimetry (507 ± 45 vs. 516 ± 55 kcal, *P* = 0.8148). Daily water throughput (litres day^−1^) was calculated using the following equation:

(3)

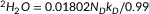




assuming H_2_O = 0.01802 litres mol^−1^ and a fractionation correction of 0.99. For a northern European recreational athlete population, the RMR (MJ day^−1^) was calculated using the Oxford equations (Henry, 2005) according to sex and age, which produced near identical values to the Müller equation for athletes using fat‐free mass (Müller et al., 2004):

(4) $$\begin{array}{ccc} Males & Age18-30 & 0.0600W+1.31H+0.473\\ & Age30-60 & 0.0476W+2.26H-0.574\\ Females & Age18-30 & 0.0433W+2.57H-1.18\\ & Age30-60 & 0.0342W+2.10H-0.0486 \end{array}$$

Pre‐row body mass (*W*) was in kilograms, height (*H*) was in metres and MJ day^−1^ was converted to kcal day^−1^ using 1 MJ = 238.847 kcal. Metabolic scope was calculated as TEE/RMR.

### 2.6 Body composition

Height and body mass (SEC‐170, Seca, Hamburg, Germany) were measured while standing. Body composition was measured using a bioelectrical impedance analyser (BIA, BodyStat Quadscan 4000, Douglas, Isle of Man, UK, manufacturer's equation) in a supine position after 15 min rest with electrodes placed in the tetra‐polar (wrist‐ankle) arrangement as per manufacturer's guidelines. The analyser measured impedance at 5, 50, 100 and 200 kHz and used the 50 Hz frequency to predict the value of total body water, fat mass and fat‐free mass. Measurements were taken in duplicate, 2 min apart and averaged.

### 2.7 Muscle thickness and architecture

Longitudinal ultrasound images were obtained at standardised muscle sites on the right side of the body. In 2020, ultrasound was taken using a 10 Hz linear transducer, using B‐mode ultrasonography (SIUI Apogee 1000 Neo, SIUI, Shantou, Guangdong, China) held vertically over the site and aligned longitudinally along the fascicle plane to allow clear detection of the superficial and deep aponeuroses of the GM and SOL. In 2021, due to technical advances, a wireless technology 7.7 Hz linear transducer, using B‐mode ultrasonography (VScan Air, GE Healthcare, Chalfont St Giles, UK) was used for all ultrasound measurements. Bland–Altman analyses of these two methods showed minimal bias (0.0021 ± 0.095 cm) and were deemed comparable. Images were captured three times, exported, and analysed using ImageJ (version 1.53e) according to Cronin (2022). Each image was analysed three times and averaged (intrasubject coefficient of variation (CV) 3.9% and experimenter CV 2.6%).

#### 2.7.1 Lower limb measurements

##### Gastrocnemius medialis and soleus

**FIGURE 2:**
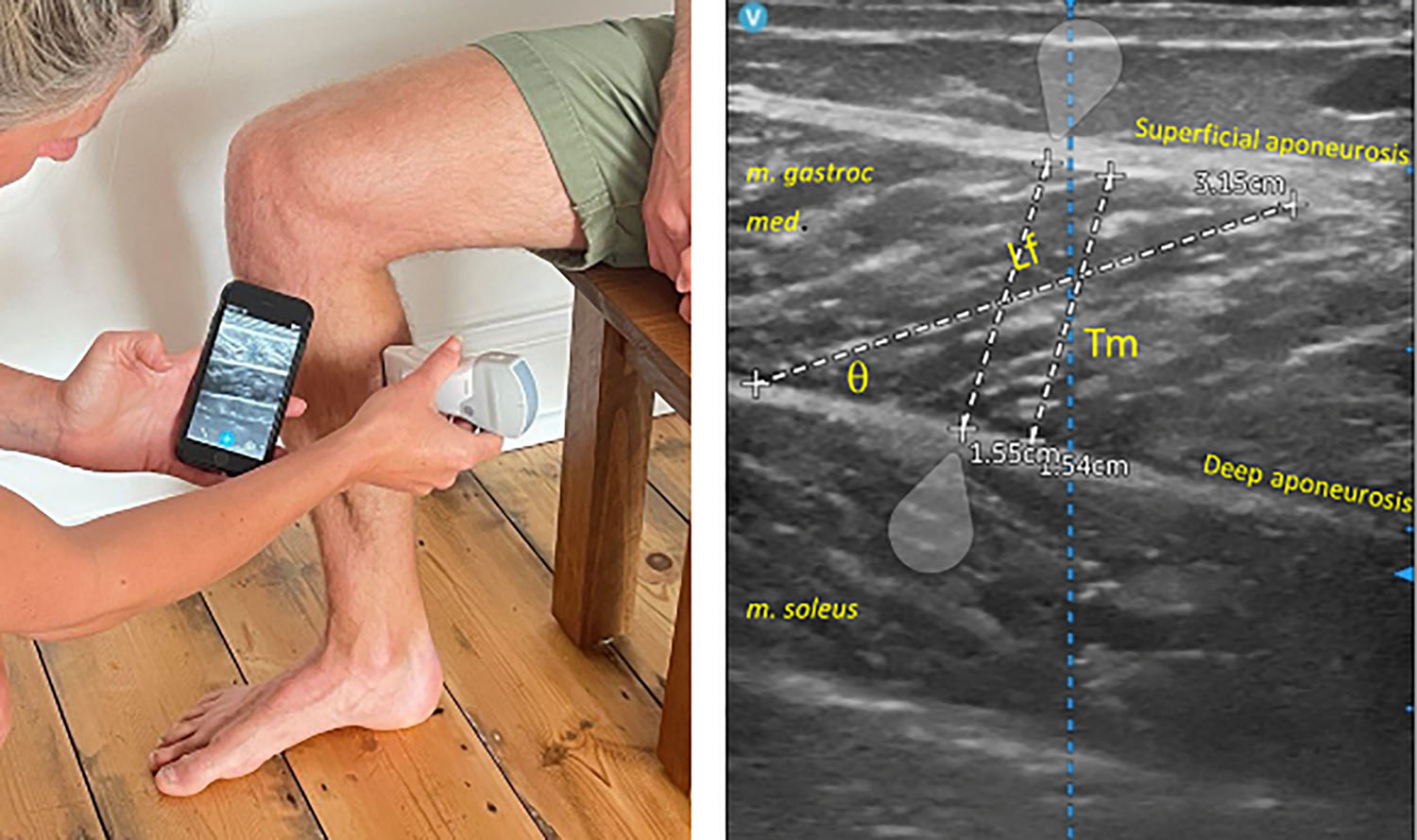
Ultrasound scan of calf in longitudinal plane (a) and muscle architectural measurements (b).

In accordance with suggested standardised protocol (Fujiwara et al., 2010) GM and SOL measurements were taken one‐third of the distance proximal to the right medial knee joint line and medial malleolus. Calf circumference was measured at this point, 1 cm above, and 1 cm below. If the calf muscle was larger either above or below, that site was used for the measurements. Exact location was recorded to enable post‐row site replication. There is evidence to suggest that atrophy is variable both within and between muscles, depending on their type and function, with maximal rates of atrophy occurring at the widest part of the muscle (Hodson‐Tole & Lai, 2019; Kilroe et al., 2020). Priority, therefore, was given to finding the location with the largest circumference. GM *T*_m_ was measured as the distance between superficial and deep aponeurosis (Figure 2) while SOL *T*_m_ was measured as the distance between deep aponeuroses and deep central structures of FDL or FHL, posterior tibial vein and artery. Fascicle length (*L*_f_) was measured on three different fascicles per image. Where *L*_f_ appeared to extend beyond the image, the line was manually extended and extrapolated. However, to minimise the error introduced by extrapolation, if the line extended to more than 50% of the visible fascicle, the measurement was discarded. PA was measured as the angle between fascicle and deep aponeuroses and was measured at three different points on each image and averaged.

##### Vastus lateralis and vastus intermedius

Longitudinal VL and VI ultrasound measurements were taken from the same site and adapted from Fischer et al. (2020) to accommodate the field environment and ensure post‐row replication. Measured in standing 50% of the distance between anterior superior iliac spine and the lateral joint line of the right knee. The ultrasound was then taken in a seated position, with hip and knee at 90° and the bare foot flat on the floor. VL *T*_m_ was measured as the distance between superficial and deep aponeurosis border with the VI. VI was measured from the deep aponeurosis of the VL and the femur.

#### 2.7.2 Upper limb measurements

##### Biceps brachaii and triceps brachaii

Upper limb ultrasound was taken standing with arms in the anatomical position. BB was measured at 50% of the distance between the right anterior acromion and the centre of the forearm antecubital crease. *T*_m_ was measured from the superficial to deep aponeurosis border with the brachiailis. TB was measured as 50% between the right posterior acromion and olecranon and *T*_m_ was measured from the superficial aponeuroses to the humerus.

##### Rectus abdominis

RA ultrasound was taken standing, measured 2 cm horizontally to the right of the naval. This was adjusted superiorly or inferiorly to ensure the muscle belly was measured and not the band of connective tissue. The exact location was recorded to ensure post‐row site replication.

### 2.8 Serum biochemical parameters

Venous blood was obtained from the cephalic or cubital forearm vein into a vacutainer containing silica to clot the blood cells and a serum separating gel to separate blood cells from plasma. Samples were left for 30 min at room temperature, centrifuged (10 min, 1,538 *g*; Hettich EBA 200) and the serum was pipetted into Eppendorf tubes and frozen, firstly at −20°C and subsequently transferred and stored at −80°C. Samples were analysed at Exeter Clinical Laboratory at Royal Devon University Healthcare Hospital for calcium, sodium, potassium, magnesium, creatinine, albumin, urea and C‐reactive protein concentrations (Cobas, Roche, Mannheim).

### 2.9 Statistical analysis

Student's *t*‐test was used to detect any sex differences in energy intake and energy expenditure. Two‐way repeated measures ANOVA (time and sex) with Bonferroni post‐hoc tests was used to determine any differences in body mass, body composition, muscle thickness and serum biochemical parameters. Linear regression was performed to investigate relationships between rate of body mass loss versus TEE, EI and muscle thickness loss, age versus TEE, EI and muscle thickness loss, and TEE and MS with time at sea. A two‐way ANOVA was used to detect time and sex differences in race duration between the 2020 and 2021 races. Statistical significance was set at *P* < 0.05. Data are reported and means ± standard deviation or means ± 95% confidence intervals.

## 3 RESULTS

In 2020, 25 rowers (3 male solo, 10 team male, 12 team female) completed the Talisker Whisky Atlantic Challenge gathering complete data sets on TEE (bar one data point), EI, body and muscle mass (Table 1). In 2021, 24 (19 team male, 5 team female) complete muscle thickness data sets were obtained. Participants' mean age, height, body mass loss, time at sea and RMR did not differ between 2020 and 2021 races. Race records from 2015 to 2022 show that the races were comparable in terms of typical race duration for both males and females (team male 41.1 ± 6.9 vs 40.8 ± 4.7 days, *P* > 0.99: team female 47.0 ± 8.4 vs 46.9 ± 3.8 days, *P* > 0.99: solo male 59.5 ± 12.7 vs 56.8 ± 11.1 days, *P* = 0.99, respectively). Rowers rowed in a continuous 2–3 h on/off pattern 24 h a day, for a minimum of 12 h per day (6 × 2 h shifts).

**TABLE 1:** Participant characteristics, body composition and serum biomarkers for male and female pre‐ and post‐row. Results are value (SD), values in brackets next to blood parameters indicate ‘normal’ ranges.

	Female (*n* = 17)		Male (*n* = 32)	
Parameter	Pre	Post	Pre	Post
Age (years)	37.3 (11.6)	–	41.5 (11.7)	–
Age Range (years)	24–58	–	29–67	–
Height (m)	1.70 (0.04)	–	1.81 (0.1)	–
Time at sea (days)	–	46.59 (3.7)	–	45.8 (7.9)
Body mass (kg)	71.6 (6.1)	64.2 (6.3)*	91.7 (11.3)	81.5 (9.5)*
RMR (kcal day^−1^) ^#^	1487 (88)	1415 (88)*	1903 (132)	1777 (112)*
Fat mass (%)^#^	26.6 (4.9)	21.6 (6.1)*	16.8 (6.4)	12.4 (6.6)*
Fat mass (kg) ^#^	19.6 (4.5)	14.6 (4.7)*	16.0 (7.3)	10.7 (6.1)*
Fat free mass (%)^#^	73.4 (4.9)	78.4 (6.1)*	83.2 (6.4)	87.6 (6.6)*
Fat free mass (kg) ^#^	53.4 (3.5)	51.9 (4.0)*	76.3 (6.6)	73.5 (7.0) *
BMI (kg m^−2^) ^#^	25.2 (2.1)	22.9 (2.0)*	27.5 (3.7)	24.9 (3.1)*
Albumin (g l^−1^) ^#^ (38–51 g l^−1^)	44.7 (3.47)	43.4 (3.5)	47.6 (2.26)	44.3 (2.41)*
Calcium (mmol l^−1^) ^#^ (2.2–2.6 mmol l^−1^)	2.3 (0.1)	2.3 (0.0)	2.3 (0.1)	2.3 (0.0)
Creatinine (μmol l^−1^) ^#^ (m 59–104 μmol l^−1^; f 44–80 μmol l^−1^)	70.9 (10.0)	73.7 (12.7)*	88.7 (13.4)	83.6 (10.2)*^†^
Potassium (mmol l^−1^) ^#^ (3.5–5.3 mmol l^−1^)	4.2 (0.1)	4.3 (0.3)	4.2 (0.3)	4.3 (0.3)
Magnesium (mmol l^−1^) ^#^ (0.7–1.0 mmol l^−1^)	0.9 (0.1)	0.8 (0.1)	0.9 (0.1)	0.8 (0.1)
Sodium (mmol l^−1^) ^#^ (133–146 mmol l^−1^)	138.2 (1.5)	139.2 (2.1)	138.6 (2.1)	138.9 (1.3)
Urea (mmol l^−1^) ^#^ (2.5–7.8 mmol l^−1^)	5.01 (1.3)	5.79 (2.35)	6.31 (1.18)^†^	6.9 (1.57)^†^
CRP (mg l^−1^) ^#^ (<5 mg l^−1^)	<5	<5	<5	<5

^#^ Parameters for 2020 cohort only (female *n* = 13, male *n* = 12). *Significantly different pre–post row, *P* < 0.05. ^†^Significantly different male vs. female, *P* < 0.05 (2‐way RM ANOVA).

### 3.1 Energy intake

**FIGURE 3:**
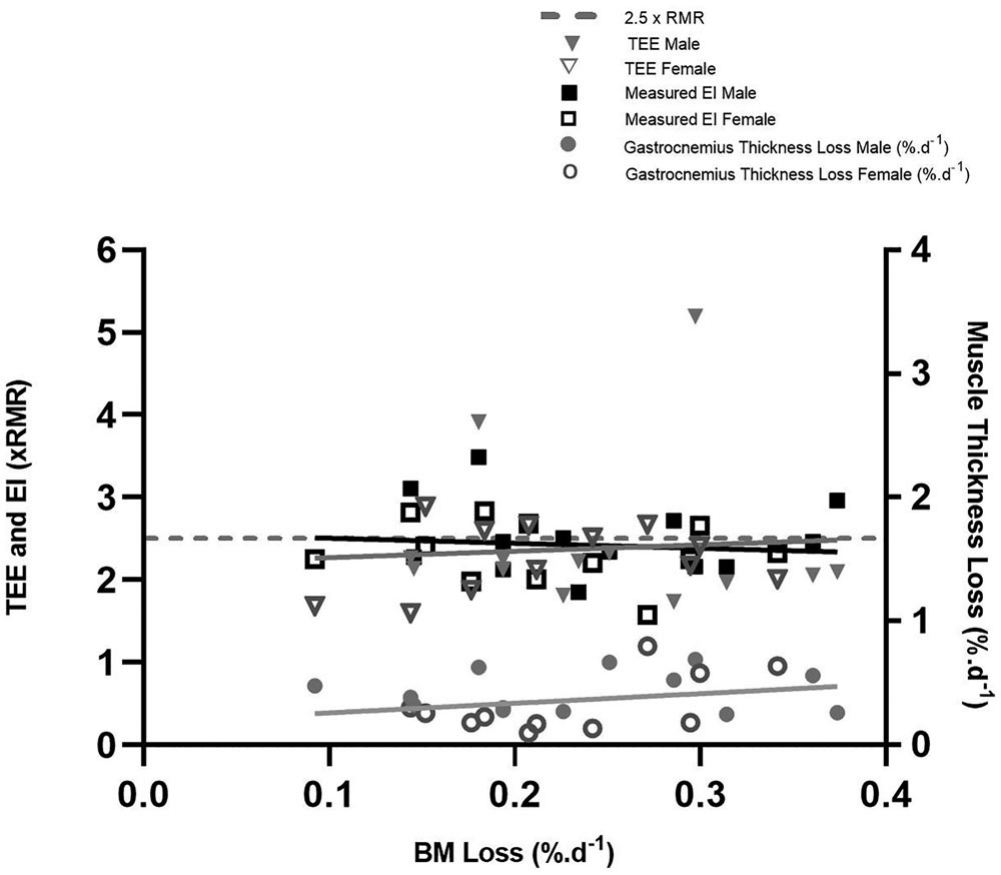
TEE (triangles, *P* = 0.7139), measured EI (squares, *P* = 0.6062) and percentage muscle (gastrocnemius) thickness loss (circles, *P* = 0.213) against body mass loss percentage per day for males (filled symbols) and females (open symbols).

**FIGURE 4:**
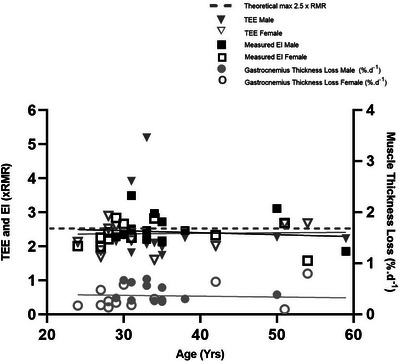
TEE (triangles, *P* = 0.9344), measured EI (squares, *P* = 0.545) and percentage muscle (gastrocnemius) thickness loss (circles, *P* = 0.750) against age for males (filled symbols) and females (open symbols).

EI measured from ration packs and from body composition (TEE—energy from body mass change) is shown in Table 2. Solo males consumed 5085 ± 1440 kcal day^−1^ whilst team males consumed 4282 ± 647 kcal day^−1^ (*P* = 0.178). Mean relative EI was 2.5 ± 0.4 × RMR (team female 2.3 ± 0.4 × RMR, team male 2.4 ± 0.3 × RMR, solo 2.9 ± 0.7 × RMR). Energy deficit was −137 ± 1694 kcal day^−1^ and −94 ± 789 kcal day^−1^ for males and females, respectively. Energy intake from body composition changes was lower than measured EI (*P* = 0.0020) at 1.7 ± 1.0 × RMR and 1.5 ± 0.3 × RMR for males and females, respectively. There was no relationship observed between rate of body mass loss and measured EI (*P* = 0.6062, Figure 3) or energy deficit (*P* = 0.6167) and no relationship between age and measured EI (*P* = 0.545, Figure 4) or energy deficit (*P* = 0.8048). For macronutrient content (Table 2) males and females were matched for carbohydrate and fat intake but males ingested greater amounts of protein than females (2.2 ± 0.43 vs. 1.5 ± 0.5 g kg day^−1^, respectively, *P* = 0.001). Rowers reported not taking any supplements in the form of energy powders or gels. Subject‐level data are provided in Table 3.

**TABLE 2:** Total energy expenditure (TEE), measured energy intake (EI) and macronutrients for females and males (solo and team combined).

	Female (*n* = 13)	Male (*n* = 12)
Energy expenditure (kcal day^−1^)	3200 (565)	4331 (1457)^†^
MS (×RMR)	2.3 (0.4)	2.5 (1.0)
EI, measured (kcal day^−1^)	3294 (502)	4468 (885)^†^
EI, measured (×RMR)	2.3 (0.4)	2.5 (0.5)
EI, TEE − body composition change (kcal day^−1^)	2080 (488)*	3024 (1561)^#*^
EI (×RMR)	1.5 (0.3)^‡^	1.7 (0.5)^‡^
Percentage of ration pack eaten (%)	76.3 (14.2)	82.6 (14.4)
Carbohydrate (g kg day^−1^)	5.8 (0.84)	5.7 (1.2)
Total energy intake (%)	46.4 (4.8)	42.0 (3.5)
Fat (g kg day^−1^)	2.4 (0.55)	2.5 (0.4)
Total energy intake (%)	42.0 (3.3)	42.1 (2.0)
Protein (g kg day^−1^)	1.5 (0.5)	2.15 (0.4) ^†^
Total energy intake (%)	11.6 (2.3)	15.9 (2.5) ^†^
Alcohol (g kg day^−1^)	0.0	0.0

^†^ Significantly different male vs. female, *P* < 0.05. ^#^ *P* = 0.054 (Student's *t*‐test) male vs. female. ^*^EI (TEE–body composition) lower than respective EI (measured), *P* = 0.0002. ^‡^EI RMR (TEE–body composition) lower than respective EI (measured), *P* = 0.002.

**TABLE 3:** Subject‐level data for body mass (BM) loss, resting metabolic rate (RMR), total energy expenditure (TEE), metabolic scope (MS), energy intake (EI), fat loss, fat free mass (FFM) loss and calf muscle thickness loss.

Participant	BM loss (kg) pre‐post (measurement taken <1 h)	BM loss (kg) (measurement taken at 24 h)	RMR (kcal day^−1^)	TEE (kcal day^−1^)	MS (TEE/RMR)	EI (kcal day^−1^)	EI (EI/RMR)	Fat loss (kg)	FFM loss (kg)	Muscle loss (%)
1	8	6.0	1694	6631	3.9	5905	3.5	5.0	1.0	−32.2
2	11.4	10.1	1584	8223	5.2	3423	2.2	6.6	3.5	−34.2
3	6.6	6.4	1410	3754	2.7	3785	2.7	5.4	1.0	−4.2
4	4.6	4.8	1410	2259	1.6	3974	2.8	3.2	1.6	−13.0
5	8.8	8.2	1344	3588	2.7	2118	1.6	6.4	1.8	−34.5
6	10.8	10.6	1359	2739	2.0	3163	2.3	4.9	5.7	−27.6
7	9.2	7.8	1301	2831	2.2	2932	2.3	5.6	2.2	−8.4
8	8	7.4	1435	3615	2.5	3171	2.2	6.2	1.2	−6.2
9	5.6	5.6	1375	3570	2.6	3895	2.8	4.2	1.4	−10.6
10	11.2	10.2	1907	4326	2.3	5928	3.1	7.7	1.9	−28.3
11	8.8	7.9	1299	3133	2.4	3454	2.7	5.8	2.1	−26.6
12	4.8	3.8	1673	3580	2.1	3812	2.3	1.5	2.3	−14.8
13	5	4.2	1452	4198	2.9	3498	2.4	5.6	0.8	−11.9
14	9.2	8.8	1653	3684	2.2	3064	1.9	2.7	6.1	6.1
15	14	13.4	1949	3832	2.0	4211	2.2	8.2	5.2	−10.0
16	14	12.8	1725	3618	2.1	5109	3.0	7.6	5.2	−10.6
17	13.6	12.0	1759	3620	2.1	4331	2.5	6.5	5.5	−22.7
18	12.2	10.2	1953	3395	1.7	5311	2.7	5.1	5.1	−21.2
19	9	7.4	1507	3206	2.1	3028	2.0	5.7	1.7	−9.0
20	7.6	5.4	1596	3003	1.9	3164	2.0	5.0	0.4	−9.5
21	3.4	1.6	1491	2514	1.7	3355	2.3	4.8	−3.0	−24.9
22	6.2	5.4	1784	4022	2.3	4394	2.5	5.3	0.1	−10.9
23	9	6.4	1929	4487	2.3	4516	2.3	4.8	1.6	−24.2
24	6	4.6	1782	3779	2.1	3787	2.1	6.0	−1.4	−10.2
25	6.6	4.0	1712	3105	1.8	4288	2.5	3.0	1.0	−9.9
mean	8.5	7.4	1603	3789	2.4	3905	2.4	5.3	2.2	−16.4
SD	3.0	3.0	212.4	1241.2	0.8	930.0	0.4	1.6	2.3	10.5

### 3.2 TEE

**FIGURE 5:**
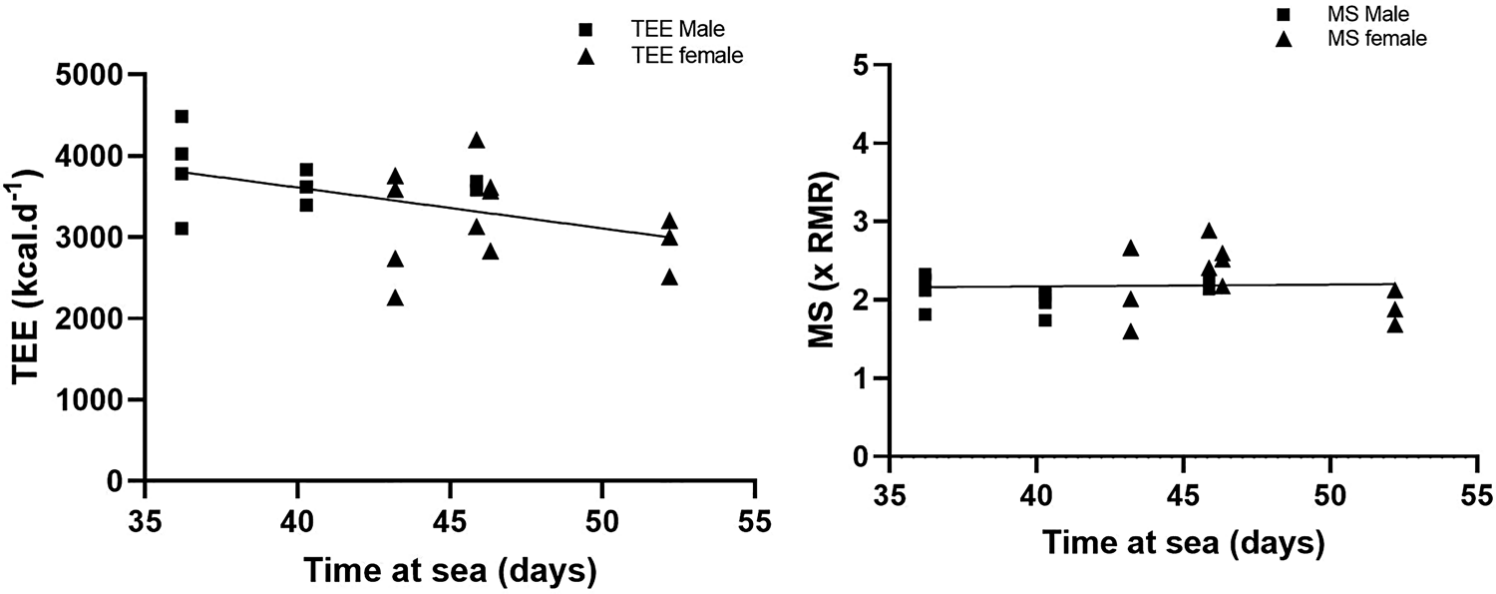
(a) TEE decreases with race duration; *P* = 0.0292. (b) When expressed relative to body size, MS does not decrease with race duration; *P* = 0.8813 for males (squares) and females (triangles). Analysis is for team rowers only.

TEE (Table 2) for solo males (6393 ± 1959 kcal day^−1^) was significantly higher (*P* < 0.001) than for team males (3712 ± 368 kcal day^−1^) and team females (3200 ± 565 kcal day^−1^). When accounting for body size, MS for male and female rowers was similar (2.5 ± 1.0 × RMR and 2.3 ± 0.4 × RMR, respectively, *P* = 0.5167) and averaged 39.8 ± 3.7 and 46.7 ± 3.4 days at sea, respectively. Solo rowers, however, had a higher MS than team rowers (3.4 ± 1.5 × RMR, *P* < 0.001) taking 58.0 ± 13.3 days. MS ranged from 1.6 to 5.2 × RMR. No relationship was observed between MS and rate of BM loss (*P* = 0.7139, Figure 3) and no relationship was observed between MS and age (*P* = 0.9344, Figure 4). Time at sea was negatively correlated with TEE (Figure 5a, *r* ^2^ = 0.22, *P* = 0.0292) but this relationship did not occur when expressed relative to RMR MS (Figure 5b, *r* ^2^ = 0.00, *P* = 0.8813). Body water turnover was 9.9 ± 2.6 litres day^−1^ for solos, which was higher than for both male (6.1 ± 2.03 litres day^−1^) and female teams (5.8 ± 1.85 litres day^−1^, *P* = 0.0060). FQ from food diaries was measured as 0.85 ± 0.01. DLW calculations are provided in Table 4.

**TABLE 4:** Doubly labelled water isotopic analyses for male and female rowers.

Sex	Age (years)	*N*_D_ (mol)	*N*_O_ (mol)	DSR	*k*_D_ (lnδ day^−1^)	*k*_O_ (lnδ day^−1^)	FQ	*r*CO_2_ (l day^−1^)	TEE (MJ day^−1^)
Male	36	2465	2328	1.027	0.121	0.158	0.84	749	18.8
Female	34	1807	1700	1.031	0.142	0.174	0.85	467	13.4

*Note*: *N*_D_ and *N*_O_, dilution spaces; DSR, dilution space ratio; *k*_D_ and *k*_O_, rates of depletion; FQ, food quotient; *r*CO_2_, rate of CO_2_ production; TEE, total energy expenditure.

### 3.3 Body composition

Body mass loss, measured pre‐race and immediately after the race was 9.6 ± 3.6 kg (10.2 ± 3.1%) and 7.2 ± 2.1 kg (10.0 ± 3.0%) for male and female rowers, respectively (Table 1). This decreased to 8.1 ± 3.5 kg (8.6 ± 3.2%) and 6.4 ± 2.3 kg (8.9 ± 3.3%) when body mass was measured again after 24 h. There was a significant time (*P* < 0.0001) and sex (*P* < 0.0001) effect and a significant sex × time interaction in absolute body mass loss, showing greater body mass loss in males than females (*P* = 0.049). However, this interaction was removed when expressed relative to pre‐row body mass, showing the greater the initial body mass the greater the body mass loss, independent of sex. There was a significant time effect (*P* < 0.0001) in absolute fat mass loss (5.3 ± 2.1kg vs. 5.0 ± 1.0 kg for males and females, respectively) but no sex effect (*P* = 0.1490) and no interaction (*P* = 0.3668). For fat free mass loss there was a significant time (*P* < 0.0001) and sex (*P* < 0.0001) effect observed (2.8 ± 2.5 kg vs. 1.5 ± 1.9 kg, for males and females, respectively) but no interaction (*P* = 0.1500) meaning whilst both lost fat free mass, males lost more but when expressed relatively, as percentage lost, there were no sex difference observed (*P* = 0.5130). Of the body mass that was lost 69.5 ± 27% and 74.4 ± 11.9% (*P* = 0.56) was fat mass loss for males and females, respectively.

### 3.4 Muscle thickness and architecture

**FIGURE 6:**
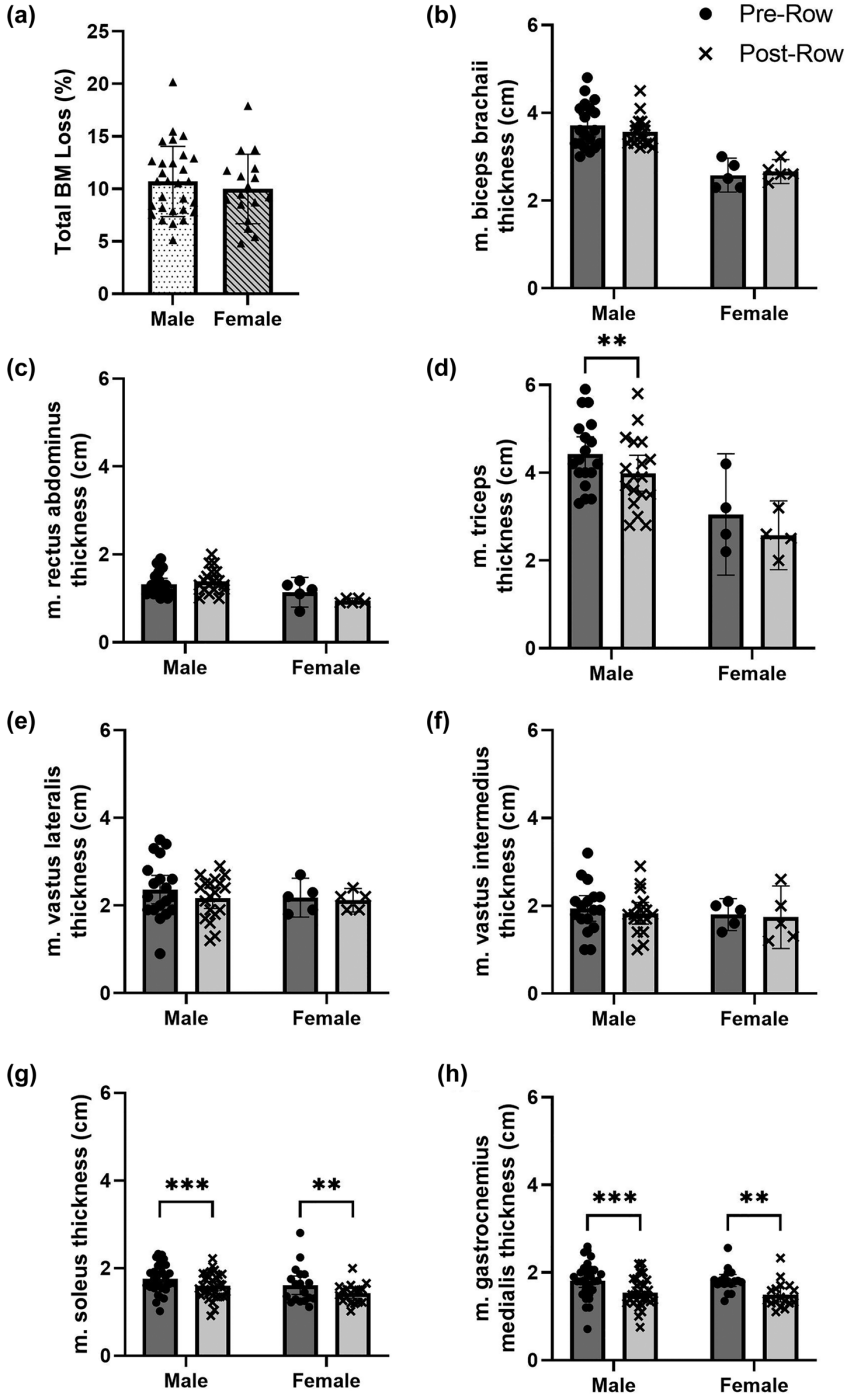
Percentage body mass loss (a) and muscle thickness changes of biceps brachaii (b), rectus abdominis (c), triceps (d), vastus lateralis (e), vastus intermedius (f), soleus (g) and gastrocnemius medialis (h), pre‐ and post‐row for males and females. **P < 0.01, ***P < 0.001. Bars are means ± 95% CI.

There was a main effect of time only on muscle thickness loss in GM (1.81 ± 0.35cm to 1.50 ± 0.31 cm, 16.1 ± 11.6%, 0.36 ± 0.26% day^−1^, *P* = 0.0002; and 1.82 ± 0.26 cm to 1.49 ± 0.3 cm, 17.8 ± 13.0%, 0.39 ± 0.3% day^−1^, *P* = 0.001), SOL (1.81 ± 0.24 cm to 1.65 ± 0.27 cm, 8.3 ± 8.7%, 0.16% day^−1^, *P* = 0.0009; and 1.63 ± 0.23 cm to 1.54 ± 0.08 cm, 4.3 ± 16.0%, 0.11 ± 0.34% day^−1^, *P* = 0.0030) and TB (4.44 ± 0.79 to 3.99 ± 0.83 cm, 9.95 ± 10.9%, 0.22 ± 0.24% day^−1^, *P* = 0.001) (Figure 6a–h). When accounting for initial muscle mass and time at sea, mean rate of gastrocnemius muscle thickness loss was 0.30 ± 0.17% day^−1^ for a total of 15.7 ± 11.4% (*P* < 0.001) and was not different between males and females (*P* = 0.8100). There was no correlation between rate of *T*_m_ loss with body mass loss (Figure 3) or age (Figure 4).

Gastrocnemius fascicle length reduced in both males and females (2.99 ± 11.6% and 3.78 ± 18.04%, respectively, *P* = 0.85) ranging from −31.77 to +42.3%. Pennation angle also reduced in both males and females (17.4 ± 17.7% and 13.4 ± 24.2%, respectively, *P* = 0.52) ranging from −53.8% to +42.5%.

### 3.5 Serum biomarkers

Pre‐ and post‐row blood serum biomarkers were within ‘normal’ ranges (Table 1) and no sex effect (*P* > 0.999 for albumin, Ca, K, Mg, Na, urea *P* = 0.6480) or time effect (*P* = 0.3110) was observed with the exception of creatinine where a significant sex × time interaction was observed (*P* = 0.0110) with creatinine decreasing for males only (*P* = 0.0320).

## 4 DISCUSSION

The present study has confirmed experimentally that there appears to be an alimentary limit to body mass maintenance during prolonged endurance ocean rowing that does not differ between males and females or with age. Direct and precise measurements of EI and EE during the final week of a transatlantic rowing race demonstrated that after approximately 6 weeks of prolonged endurance exercise, daily EI was 2.5 and 2.3 × RMR, matching TEE of 2.5 ± 1.0 and 2.3 ± 0.4 × RMR, for males and females, respectively. As expected, males and females both lost ∼10% body mass over the duration of the event, predominantly from fat and inactive muscle mass.

### 4.1 Energy intake

EI calculated from doubly labelled water measurements of TEE during previous extreme endurance events (Thurber et al., 2019) has been shown to be ∼2.5 × BMR and, thus, the theoretical alimentary limit to EI. These studies did not appear to supplement with carbohydrate energy drinks or gels. EI measured from rower ration packs in the present study supports this. Despite there being greater than 3 × RMR food available, rowers consistently and voluntarily consumed 2.3–2.5 × RMR, which was not different for sex or age. Rowers reported not supplementing with energy drinks or gels. Similar findings were recorded from dietary recall, during a 6‐week Antarctic crossing (2.7 × RMR and 2.4 × RMR EI for males and females, respectively), where it would have been advantageous to consume more food in order to drag less weight (Hattersley et al., 2020). Older cyclists (> 65 years) whose food consumption was observed and weighed by scientific staff, reported consuming 2.7 × BMR of food during a 14 day, 2706 km expedition, which increased to 3.2 × BMR with energy drinks and gel supplementation (Rosenkilde et al., 2015). Indeed, professional cyclists are able to raise their EI to 4.6 × RMR with energy drink supplementation (Westerterp et al., 1986), presumably due to supplements allowing rapid and maximal substrate delivery for oxidation to help meet the increased energy demand (Brouns et al., 1989). However, whilst this helps explain increased energy delivery rates, the reason for increased total *voluntary* food intake is not known. The additional 0.5 × BMR EI from supplemental carbohydrate in the study of older cyclists was still insufficient to offset negative energy balance and the authors reported a ‘profound counteracting physiologic stimulus inhibiting increases in EI, despite the presence of increased motivation to eat’ (Rosenkilde et al., 2015). During extreme overfeeding (Pasquet et al., 1992) sedentary males have ingested up to 3.4 × RMR (unsupplemented) but available energy (after excretion and vomit) was only 2.4–2.7 × RMR. Together this suggests the limit is at the level of digestion and absorption and related to body size.

Thus, having measured EI in a non‐elite endurance race we have shown the non‐supplemented alimentary limit to be 2.3–2.5 × RMR but suggest the term ‘maximal sustainable’ EI rather than limit, given the possibility of being able to increase it further with supplementation of simple carbohydrates.

One important point to note is that EI calculated from body mass loss in the present study was lower than EI measured from ration packs. This could be due to uneaten food not being saved, energy absorption being lower than anticipated due to competing demands of rowing or due to the overestimation of body mass loss in the calculation. TEE and EI were closely matched in the final week suggesting minimal body mass loss. To incur a 10% body mass loss it is likely that TEE was higher in the initial stages of the race and decreased with race duration. Indeed, this has been shown previously (Thurber et al., 2019) where efforts to reduce the deficit have induced a metabolic adaptive response constraining TEE, reducing RMR, or some combination of responses from all components of energy expenditure (Dolan et al., 2023). Using mean daily body mass loss calculated from overall body mass loss would therefore have overestimated true final week body mass loss resulting in an EI lower than that from ration packs. Unfortunately, it was not possible to measure TEE across the entire race or weigh rowers at sea.

### 4.2 Energy expenditure

MS was similar in male and female rowers and lower than expected. The negative curvilinear relationship between TEE and event duration proposed by Thurber et al. (2019) suggests that events of 46 days could theoretically sustain an MS of 3.8–4.6 × RMR and thus rowers may not have reached their energy expenditure limit. It is possible TEE was underestimated as RMR has been shown to reduce by ∼70–100 kcal day^−1^ beyond that accounted for by FFM loss alone, in individuals subjected to 1 week 50% overfeeding followed by 3 weeks calorie restriction (−50%) losing ∼6 kg (M‡uller, Enderle et al. 2015). RMR calculated from equations in the present study, using height and body mass, would therefore overestimate RMR and underestimate TEE by ∼0.1–0.2 × RMR. In addition, FQ reflects respiratory quotient (RQ) only in energy balance and decreases with calorie restriction (Müller, Enderle et al. 2015). Using FQ in TEE calculations in the present study may further underestimate TEE. However, the minimal final week deficit in rowers suggests adaptation has occurred and RQ may again reflect FQ, but magnitude and time course of these changes is difficult to quantify. TEE, however, is unlikely to have reached the theoretical limit of 3.8–4.6 × RMR.

The TEE–duration curve is based on a mix of study populations ranging from elite athletes to polar trekkers, the latter of whom also contend with thermoregulatory mechanisms that influence TEE. This likely shifts the curve to the right and overestimates MS for non‐elite athletes in temperate or hot climates. It also assumes equal levels of motivation and effort as a true MS requires continuous maximal effort, which may be different between elite athletes, military personnel, recreational expeditioners and ocean rowers. This is demonstrated by rowers in the present study having a shorter duration but a lower or similar MS to an Antarctic expedition lasting 67 days (3.15 × RMR) or 61 days (2.8 × RMR) Hattersley et al., 2020). To our knowledge, the study of participants in the transcontinental running race across the USA is the only other event to have included a female endurance athlete, with a TEE measured after week 1 being 4.3 × RMR (the participant pulled out after week 8 due to injury; Thurber et al., 2019). Interestingly, the mean MS for the five males at week 1 was also 4.3 × RMR (range 3.2–4.4 × RMR) dropping to 3.1 × RMR after 20 weeks, suggesting firstly, no sex difference in capacity to generate high MS, supporting the findings presented here, and secondly, TEE was higher earlier in the race. There was also no observable correlation of MS with age in the present study.

Of the four rowers that were over 50 years old, only one of them (male) was under the mean MS (range 2.6–3.4 × RMR). TEE in six older non‐elite cyclists was measured to be 4.0 ± 0.1 × BMR during a 14 day, 2706 km expedition (Rosenkilde et al., 2015), again suggesting MS is similar between older and younger athletes up to the age of 61 years old. This is surprising given the known decline in exercise performance (Maharam et al., 1999) with age, but recent evidence shows daily energy expenditure remains stable in adults up to 60 years (Pontzer et al., 2021). Combined this sheds new light on the potential capacity of older persons to perform long‐duration low‐intensity exercise. However, there are so few studies on older people in long‐duration events that drawing conclusions remains challenging.

### 4.3 Body composition

All the participants lost ∼10% body mass. Of the body mass lost there were no differences between males and females in loss of FFM (36% and 26%, respectively) and FM (64% and 74%, respectively) and this is consistent with body composition changes in the Antarctic expeditions, which showed that of the 8% body mass lost in females, FFM was 33% and FM was 74%, while males gained 15% FFM (although on closer inspection this looks to be skewed by one participant who gained 5 kg in FFM; Hattersley et al., 2019). Similarly, of the 4% body mass lost in the race across the USA, FFM was only 23% (Thurber et al., 2019). These values are similar to when losing weight using dietary restriction alone (FFM 20–30% and FM 70–80% of body mass loss) instead of dietary restriction combined with exercise (FFM 11% and FM 89% of body mass lost) (Weinheimer et al., 2010) as might be expected. The greater FFM loss observed in the present study is difficult to reconcile given that around 12 h of rowing was performed per day, but could be due to exercise intensity.

Ocean rowing is low intensity, long duration exercise vastly different in technique to an Olympic style 2 km race, using predominantly body and arms and minimal leg drive to move a ∼1.2 tonne boat with wind and wave assistance. Finding studies that report intensity, heart rate and substrate oxidation rates during high TEE and high volumes of exercise similar to ocean rowers is rare, which makes comparisons difficult. However, studies with lower TEE, dietary restriction plus exercise have shown in middle aged women that moderate intensity exercise (45–50% of maximal oxygen consumption) on a treadmill 3 days week^−1^ for 20 weeks was sufficient to reduce FFM loss (28%) compared with diet restriction alone (36%) (Nicklas et al., 2009), suggesting even at low intensity muscle can be preserved. When using the more precise ultrasound measurement of muscle thickness, we showed both males and females atrophied in the same muscle groups, with the greatest loss measured in the calf (15.63 ± 10.1% and 9.8 ± 11.5% for gastrocnemius and soleus, respectively) but maintained in the biceps brachaii and rectus abdominus. Although load was not measured, it is speculated that not standing up for the majority of the crossing and minimal leg drive meant a reduction in load to these locomotor muscles. Indeed, the magnitude of calf muscle loss is similar to 15–20% losses observed in male participants after 45 days of bed rest lying with 6° head down tilt (Rittweger et al., 2005). On the other hand, the constant rocking of the boat and the rowing lay‐back action (a potent stimulator of eccentric contraction of rectus abdominus) would likely have produced a low‐load through the trunk and the observed maintenance of mass. Indeed, rectus abdominus cross‐sectional area increased during 56 days of 6° head down tilt (Hides et al., 2007), presumably from overactivity whilst lying. We also know rowers consumed sufficient protein (2.2 and 1.5 g kg^−1^ day^−1^ for males and females, respectively) to respond to a mechanical stimulus (Longland et al., 2016). It would appear that the mean FFM changes masked the nuance of a non‐uniform dimorphic response between loaded and unloaded muscles with the majority of loss coming from the lower limbs.

In conclusion, this is the first study to show that during a prolonged endurance, ocean rowing race, over an average of 46 days, there appeared to be a maximal sustainable EI of around 2.5 × RMR, which met the final week demand of 2.5 × RMR. Given the 10% body mass loss throughout the race, EI was not able to meet the total energy demand which supports current alimentary theory with implications for constrained energy theory. Mass was lost mostly from fat, with a concurrent dimorphic muscular adaptive response favouring ‘loaded’ trunk and arm muscle, and atrophy of ‘unloaded’ calf muscles. There were no differences in the relative adaptive response of TEE, EI, body mass loss and muscle loss between males and females, and there was little effect of age. These findings could have important implications beyond extreme endurance events such, critical illness, spaceflight and ageing.

## AUTHOR CONTRIBUTIONS

This study was conducted abroad as part of Public Health and Sport Sciences, Medical School, University of Exeter. Conception, acquisition, analysis or interpretation of data: R.W., F.S. and R.I. Drafting of the work or revising it critically for important intellectual content: R.W., F.S., B.W. and R.I. Funding acquisition: R.W. and F.S. Supervision: F.S., B.W. and R.I. All authors approved the final version of the manuscript and agree to be accountable for all aspects of the work in ensuring that questions related to the accuracy or integrity of any part of the work are appropriately investigated and resolved. All persons designated as authors qualify for authorship, and all those who qualify for authorship are listed.

## CONFLICT OF INTEREST

The authors declare no conflicts of interest.

## Data Availability

Data can be made available upon reasonable request by contacting Professor F. B. Stephens at f.b.stephens@exeter.ac.uk.
